# Increased serum B‐cell maturation antigen levels evaluated with an Elecsys‐based serum B‐cell maturation antigen assay have a negative prognostic value in patients with newly diagnosed multiple myeloma

**DOI:** 10.1002/jha2.889

**Published:** 2024-12-09

**Authors:** Evangelos Terpos, Ioannis Ntanasis‐Stathopoulos, Panagiotis Malandrakis, Despina Fotiou, Magdalini Migkou, Evangelos Eleutherakis‐Papaiakovou, Foteini Theodorakakou, Maria Roussou, Maria Gavriatopoulou, Meletios A Dimopoulos, Efstathios Kastritis

**Affiliations:** ^1^ Department of Clinical Therapeutics School of Medicine National and Kapodistrian University of Athens Athens Greece

**Keywords:** B‐cell maturation antigen, BCMA, multiple myeloma, overall survival, prognosis, progression‐free survival

## Abstract

Background: Serum B‐cell maturation antigen (sBCMA) levels have emerged as a potential biomarker for disease monitoring in multiple myeloma (MM) with prognostic value.

Methods: Herein, we evaluated the sBCMA levels in 166 patients with newly diagnosed MM with an Elecsys‐based sBCMA assay.

Results: Increased sBCMA levels at diagnosis were correlated with inferior survival outcomes in terms of both progression‐free and overall survival. In a subset of patients with available samples at the time of disease progression, there was a trend for decreasing sBCMA values.

Conclusion: Sequential evaluation of sBCMA in prospective studies will determine the value of incorporating sBCMA measurement in clinical practice.

## 1 INTRODUCTION

B‐cell maturation antigen (BCMA) has emerged as a key target in the current therapeutics of multiple myeloma (MM). Several anti‐BCMA immunotherapeutic approaches including antibody‐drug conjugates, bispecific T cell engagers, and chimeric antigen receptor T cell therapy have improved patient outcomes and are under further clinical development. BCMA is a surface marker belonging to the tumor necrosis factor superfamily and is found on the surface of differentiated B‐cells including plasma cells. Surface BCMA is cleaved by the gamma‐secretase complex and soluble BCMA is subsequently released in serum. Serum BCMA (sBCMA) levels have emerged as potential biomarkers for disease monitoring with prognostic value [1].

## 2 METHODS

The aim of the present study was to evaluate the sBCMA distribution with an Elecsys^‐^based sBCMA assay (Roche Diagnostics International Ltd) and its potential prognostic role in populations of patients with MM and smoldering MM (sMM) with various demographic and disease characteristics. The Elecsys‐based sBCMA is a quantitative serologic, two‐incubation‐step assay (for research purposes) using the sandwich test format (total assay time of 18 min) for the detection of sBCMA in human serum on cobas immunoassay analyzers. The archival serum samples and de‐identified patient data were provided by a single institution. Patients had provided written informed consent. Patients with sBCMA values outside of the measuring range (1.2–900 ng/mL) were excluded from the analysis. The study was approved by the institutional review board of Alexandra General Hospital (375/210 519) and it was conducted according to the Declaration of Helsinki and its future amendments.

## 3 RESULTS

Overall, the current analysis included 166 patients diagnosed from 2018 to 2020 with a median follow‐up of 38 months. The median age was 67 years (range 50–93 years), whereas 101 (61%) were males. Among them, 122 patients were diagnosed with symptomatic MM and 44 patients with sMM. Among patients with a symptomatic disease requiring therapy, 29 (24%) received daratumumab‐based regimens, 80 (66%) received bortezomib‐based regimens, 12 (10%) received lenalidomide‐only based regimens and one patient received cyclophosphamide and dexamethasone. At best response, 38 patients (31%) achieved (stringent) complete response (sCR/CR), 43 patients (35%) achieved very good partial response, 30 patients (25%) achieved partial response (PR), and 11 patients (9%) had no response. Regarding the risk stratification, 37 patients (30%) were ISS 1, 44 patients (36%) were ISS 2, and 41 patients (34%) were ISS 3. Furthermore, 26 patients (21%) had IgA myeloma, 76 patients (62%) had IgG myeloma, 11 patients (9%) had kappa light chain myeloma, five patients (4%) had lambda light chain myeloma, and four patients (3%) had other myeloma subtypes.

At diagnosis, the baseline mean sBCMA value was 162 ng/mL (standard deviation [SD] 169) for patients with MM and 19.4 ng/mL (SD 16.5) for patients with SMM. The log sBCMA values were 4.56 ng/mL (SD 1.11) and 2.75 ng/mL (SD 0.62) for MM and sMM, respectively. Regarding patients with symptomatic MM, there was a trend towards higher sBCMA at baseline in IgA compared to IgG subtypes, although the difference was not statistically significant. More specifically, patients with IgA MM (*n* = 26) had a mean sBCMA value of 280 ng/mL (SD 193), whereas patients with IgG MM (*n* = 76) had a mean sBCMA value of 121 ng/mL (SD 127). In addition, there was no meaningful association between sBCMA baseline values and best response during first‐line treatment.

The baseline mean sBCMA value was lower in patients without documented disease progression (103 ng/mL, SD 107) compared with patients who had one (200 ng/mL, SD 194) or two (198 ng/mL, SD 185) disease progressions during the follow‐up period. In a subset of patients with MM, there were available data at the time of first (*n* = 68) and second (*n* = 20) disease progression. At first disease progression, the mean sBCMA value was 102 ng/mL (SD 148), whereas at second disease progression, the mean sBCMA value was 169 ng/mL (SD 275). There was a general trend towards decreasing sBCMA values from baseline to first disease progression (estimated mean difference 45%, absolute mean difference −82.9 ng/mL, 95% confidence interval [95%CI]: −118 to −47.8 ng/mL, and *p* < 0.0001). In general, the results were consistent among the risk stratification subgroups based on ISS staging (for ISS 1 the estimated mean difference was 62.0%, which corresponds to a 38.0% decrease from baseline, *p* = 0.1808, for ISS 2 the estimated mean difference was 47.2%, which corresponds to 52.8% decrease from baseline, *p* = 0.0187, and for ISS 3 the estimated mean difference was 31.1% which corresponds to 68.9% decrease from baseline, *p* < 0.0001). The sample size was rather small for the second disease progression timepoint (the estimated mean difference of 56.1% which corresponds to a 43.9% decrease from baseline, *p* = 0.028).

**FIGURE 1:**
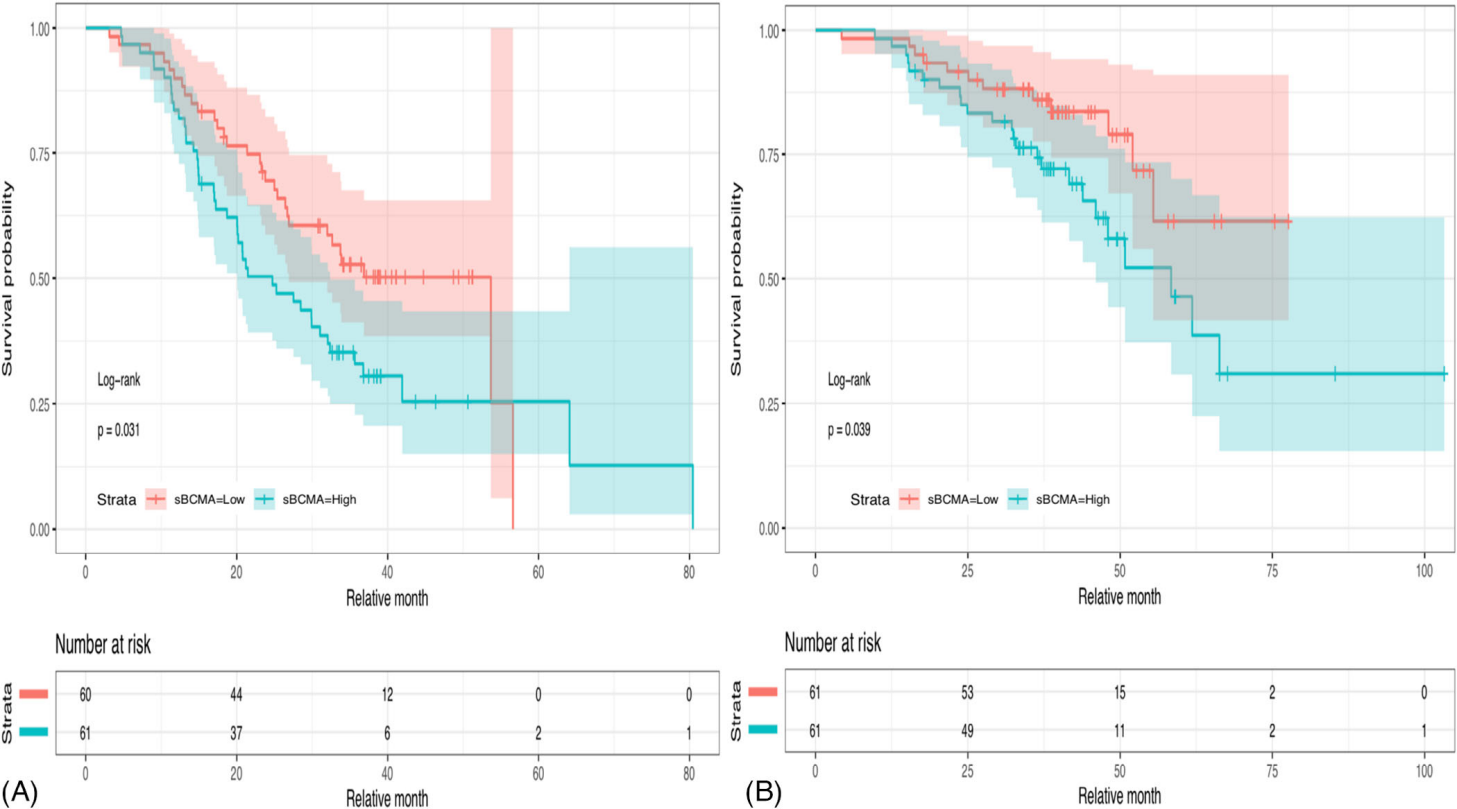
Kaplan‐Meier curves for the probability of progression‐free survival (PFS) (A) and overall survival (OS) (B) for patients with multiple myeloma (MM) according to the serum B‐cell maturation antigen (sBCMA) levels at diagnosis. sBCMA concentration is considered High when it's equal to or above 113 ng/mL (median of baseline sBCMA values). Low sBCMA concentration is considered to be below 113 ng/mL.

Furthermore, patients with symptomatic MM were categorized as low (*n* = 61) or high expressors (*n* = 61) based on sBCMA expression at baseline; low expressors had baseline sBCMA values below 113 ng/mL (median) and high expressors had baseline sBCMA values ≥ 113 ng/mL. The median progression‐free survival (PFS) was 24.7 months (95%CI: 20.1–32.4) for high expressors and 53.7 months (95%CI: 26.9 to not reached) for low expressors. Therefore, low sBCMA expressors at diagnosis had a PFS advantage over high sBCMA expressors (hazard ratio [HR] 1.67, 95%CI: 1.04–2.67, and log‐rank *p* = 0.031; Figure 1A). In the subgroup analysis according to ISS, a significant association became evident only for patients with ISS 3 (HR 2.24, 95%CI: 1.12–4.48, *p* = 0.023, high vs. low expressors).

Interestingly, baseline sBCMA levels were correlated with overall survival (OS), as well. High expressors had a median OS of 58.4 months (95%CI: 46 to not reached) compared with low expressors for whom the median OS was not reached. Therefore, there was a trend for an adverse prognostic role of high baseline sBCMA values in terms of OS (HR 2.05, 95%CI: 1.02–4.11, log‐rank *p* = 0.039, Figure 1B).

## 4 DISCUSSION

The results of our study coincide with previous literature in the field supporting a key role of sBCMA as a biomarker for patients with MM. We found that patients with asymptomatic disease have lower levels of sBCMA than those with symptomatic MM. Similarly, Sanchez et al have reported increased levels of sBCMA among MM patients compared to patients with monoclonal gammopathy of undetermined significance (MGUS) [2]. Interestingly, Visram et al have indicated that sBCMA levels are higher among patients with MGUS or sMM who eventually progress to symptomatic MM compared to those who remain in the precursor state. The longitudinal assessment of sBCMA levels enabled the early identification of those patients with gradually increasing sBCMA values before the establishment of disease transformation to symptomatic MM [3].

Although patients with MM have increased sBCMA levels at diagnosis, a drop becomes evident as soon as the disease responds to treatment, whereas there are preliminary data suggesting that the magnitude of the decline may be correlated with PFS, as well [2, 4]. The role of sequential monitoring of sBCMA levels is clearly depicted in disease monitoring in patients receiving anti‐BCMA treatment. Shen et al showed that the administration of the anti‐BCMA chimeric antigen receptor T‐cell ciltacabtagene autoleucel leads to a firm decrease in sBCMA levels at least for the first 3 months after the infusion. Importantly, a subsequent increase in sBCMA among responders preceded the disease relapse according to the IMWG criteria [5]. Similar results of an early increase in sBCMA before documented myeloma progression have been also reported with non‐BCMA targeted therapies [6].

In our study, a prognostic role of sBCMA at MM diagnosis emerged for both PFS and OS. Increased sBCMA levels have been also correlated with adverse survival outcomes in previous studies [1, 2]. A study by Bujarski et al showed that this effect may be extended to patients with relapsed/refractory MM (RRMM) based on sBCMA at the time of disease relapse, as well [6]. The timing of achieving the nadir in sBCMA levels did not seem to have an impact on patient outcomes, whereas an increase in sBCMA was predicted for inferior PFS even in patients with RRMM [6].

The main limitation of our study is the limited number of patients with sequential samples available at the time of disease relapse, in addition to the absence of samples at the time of best response. Therefore, several comparisons may have been underpowered to detect significant differences between the examined subgroups. Although there are also other assays available for sBCMA measurement, the Elecsys‐based sBCMA assay applies a robust methodology, is time‐sparing, and is convenient to use. Currently, there is no established “gold standard” method for evaluating sBCMA levels and a comparative study of different assays would be valuable. Taking into consideration the promising role of sBCMA examination in MM monitoring and its potential widespread application in the future, the availability of several assays will assure clinicians access to these tools. sBCMA levels may be proven particularly useful for the monitoring of patients with oligosecretory or non‐secretory MM since they do not correlate with the monoclonal immunoglobulin, whereas they may reflect the disease burden. However, before the prime time of sBCMA in MM therapeutics, there are several challenges to overcome which mainly pertain to the standardization of techniques, the definition of cut‐off values, and the independent validation of the study results.

In conclusion, patients with symptomatic MM have higher sBCMA values at diagnosis compared with those with asymptomatic disease. Importantly, those with MM and high baseline sBCMA levels seem to have a dismal prognosis in terms of both PFS and OS compared with those with low sBCMA levels. Sequential evaluation of sBCMA in prospective studies will determine the value of incorporating sBCMA measurement in clinical practice.

## AUTHOR CONTRIBUTIONS

Evangelos Terpos, Ioannis Ntanasis‐Stathopoulos, Panagiotis Malandrakis, Despina Fotiou, Magdalini Migkou, Evangelos Eleutherakis‐Papaiakovou, Foteini Theodorakakou, Maria Roussou, Maria Gavriatopoulou, Meletios A Dimopoulos, and Efstathios Kastritis performed the research and provided data. Evangelos Terpos and Ioannis Ntanasis‐Stathopoulos wrote the first draft. All authors critically reviewed the manuscript and agreed on the final version.

## CONFLICT OF INTEREST STATEMENT

The authors declare no conflict of interest. COBAS and ELECSYS are trademarks of Roche.

## ETHICS STATEMENT

The authors have confirmed ethical approval statement is not needed for this submission.

## PATIENT CONSENT STATEMENT

The authors have confirmed patient consent statement is not needed for this submission.

## CLINICAL TRIAL REGISTRATION

The Elecsys‐based sBCMA assay is used for research purposes only and is not approved for clinical use.

## Data Availability

The data that support the findings of this study are available from the corresponding author upon reasonable request.
